# A cross-sectional analysis of AI readiness and attitudes among nurses in resource-limited Chinese county hospitals

**DOI:** 10.3389/fdgth.2026.1778627

**Published:** 2026-03-05

**Authors:** Ming Yu, Rong Yu, Mengjia Zhou, Xiaoli Fan, Ronghui Geng, Jing Ji, Suping Cai, Lili JIang, Lingling Jiang

**Affiliations:** Department of Nursing, Affiliated Rudong Hospital of Xinglin College, Nantong University, Nantong, Jiangsu, China

**Keywords:** AI attitudes, China, clinical nurses, county hospital, influencing factors

## Abstract

**Aim:**

To investigate the current situation of clinical nurses' attitudes towards artificial intelligence in county hospitals and analyze its influencing factors, so as to provide a reference for promoting the application of artificial intelligence technology in the field of primary medical care.

**Design:**

A descriptive, cross-sectional study.

**Methods:**

A total of 449 clinical nurses from a Chinese county-level B-level hospital in Nantong City were selected from August to September 2025 by convenience sampling, and the general information questionnaire, the Attitude Scale for the Application of Artificial Intelligence Technology in Nursing, the Artificial Intelligence Literacy Scale and the Change Fatigue Scale were used to investigate the influencing factors.

**Results:**

The total score of clinical nurses’ attitudes toward AI was 45.17 ± 2.38, indicating a moderate level. Multiple linear regression analysis identified age, participation in AI-related training, education level, number of monthly night shifts, change fatigue, and total AI literacy score as significant determinants of AI attitudes (all *P* < 0.05). Collectively, these factors accounted for 60.6% of the total variance in AI attitude scores.

**Conclusion:**

The attitude of Chinese county-level clinical nurses towards AI is at a moderate level and is influenced by multiple modifiable factors. To enhance AI acceptance and facilitate its integration into primary care, we recommend implementing targeted AI training programs, improving AI literacy, optimizing scheduling to reduce night shift burdens, and proactively managing change fatigue.

## Introduction

The deep integration of Artificial Intelligence (AI) into the healthcare sector is profoundly reshaping nursing practice, presenting a dual landscape of significant opportunities and formidable challenges (1). On one hand, AI-driven technologies hold immense potential to enhance diagnostic accuracy, streamline clinical workflows, alleviate administrative burdens, and facilitate the development of personalized care plans. On the other hand, their adoption raises critical concerns regarding data privacy and security, algorithmic bias, the redefinition of clinical responsibilities, and the potential erosion of the humanistic elements fundamental to nursing care. As the primary end-users and core facilitators of these technologies at the bedside, nurses' attitudes, perceptions, and readiness are pivotal determinants influencing the successful implementation and sustained effectiveness of AI in clinical settings. A growing body of international evidence suggests that nurses often hold ambivalent attitudes toward AI, characterized by a recognition of its efficiency-enhancing capabilities coexisting with profound apprehensions about ethical dilemmas, patient safety, and the evolving nature of their professional roles (2). While this topic is gaining traction globally, research within the Chinese context remains in its nascent stages. Furthermore, the existing limited body of Chinese literature predominantly focuses on nurses working in resource-rich, top-tier tertiary hospitals (3, 4). This focus creates a significant knowledge gap, leaving the attitudes of nurses in county-level hospitals—the backbone of China's primary healthcare system—largely unexplored and poorly understood.

County-level hospitals serve as crucial hubs within China's primary healthcare system, acting as a vital bridge connecting urban tertiary care centers and rural health clinics. The nursing workforce within these institutions is the cornerstone for realizing the national strategy of “sinking” high-quality medical resources to the grassroots level, thereby empowering and strengthening the entire primary care network (5). Consequently, investigating this specific cohort's authentic attitudes toward AI, along with the underlying determinants, carries substantial and immediate implications. First, it addresses a critical evidence gap regarding technology acceptance at the primary care level. The findings can directly inform the development of targeted training programs, shape supportive policies for equitable technology distribution, and guide the user-centric design of AI tools tailored to the unique contexts of county hospitals. Ultimately, understanding and addressing the nurses’ perspectives is a fundamental step toward ensuring that the integration of AI technologies is not only technologically successful but also equitable, accessible, and sustainable, truly enhancing the quality and efficiency of care for the vast population reliant on these essential institutions.

While the aforementioned attitudes are critical, they do not form in a vacuum. Evidence consistently highlights that a nurse's artificial intelligence literacy—comprising the awareness, understanding, and ethical evaluation of AI—and the receipt of structured AI training are pivotal factors that directly shape their competence and confidence in using these technologies (6). However, a crucial question remains unanswered: precisely how do these foundational factors—AI literacy and training—influence and potentially reshape the often-ambivalent attitudes held by nurses in county-level hospitals? The specific pathways and the relative strength of their impact within the unique, resource-constrained context of primary care are not yet well-defined. This gap in understanding the mediating mechanisms between these enabling factors (literacy/training) and the resulting attitudes significantly hinders the development of evidence-based, highly targeted interventions aimed at fostering AI acceptance in these vital healthcare settings.

Therefore, this study targets clinical nurses in county-level hospitals with a threefold objective: first, to investigate the current status of their attitudes toward AI; second, to systematically analyze the key influencing factors, particularly the roles of AI literacy, training, and change fatigue, guided by technology acceptance frameworks; and ultimately, to provide a robust empirical basis for formulating tailored intervention strategies. The findings are expected to bridge the identified knowledge gap and offer actionable insights to facilitate the smooth, equitable, and effective integration of AI technologies within these essential primary care institutions.

## Methods

### Study design

This is a descriptive, cross-sectional study. Its methodological component involves the application and context-specific assessment of validated scales—the Attitude Scale Towards the Use of Artificial Intelligence Technologies in Nursing (ASUAITIN), the Artificial Intelligence Literacy Scale (AILS), and the Change Fatigue Scale—within the setting of county-level hospitals in China.

### Participants and settings

In China, healthcare resources are distributed hierarchically: major urban areas host the highest-level medical institutions for their regions, while each county also has its own leading hospital within the local tier. Hospitals are classified into three levels (primary, secondary, tertiary) based on their service capacity and resources, with “tertiary” representing institutions that demonstrate the highest standards in clinical service, technical capability, and teaching and research. Each level is further subdivided into three grades—A, B, and C—with Grade A being the highest and predominantly located in urban cores. This cross-sectional study was conducted between August and September 2025 in a county-level tertiary B hospital in Nantong City, Jiangsu Province, China. Equipped with the most advanced medical facilities within the county, this hospital serves as a key clinical and technical hub supporting the local primary healthcare system, making it an ideal setting for investigating the attitudes of frontline nurses. Convenience sampling was used to recruit clinical nurses for the survey.

Inclusion criteria were: (1) being a registered clinical nurse; (2) having at least six months of clinical nursing experience; (3) having experienced the hospital relocation process; and (4) providing informed consent. Exclusion criteria were: (1) nurses undergoing further training or internships; (2) non-clinical staff (e.g., nursing department administrators).

The sample size was calculated using the formula *n* = [(Zα/2 × CV)/ε]^2^, where Zα/2 = 1.96, CV = SD/mean, and ε = 2%. Based on a previous study by Hu et al. (4), CV was estimated as 0.1556. Accounting for a 20% invalid response rate, the minimum required sample size was 274. Ultimately, 449 valid responses were included in the final analysis, exceeding the calculated minimum requirement.

### Demographic and professional information questionnaire

A self-developed Demographic and Professional Information Questionnaire was used to collect demographic and professional characteristics of the clinical nurses. The collected data included age, gender, educational level, professional title, frequency of monthly night shifts, and whether they had participated in AI-related training.

### Attitude scale towards the use of artificial intelligence technologies in nursing (ASUAITIN)

The Attitude Scale Towards the Use of Artificial Intelligence Technologies in Nursing (ASUAITIN), originally developed by Dilek Yılmaz et al. (7), was used to assess nurses' attitudes toward AI technology application. The Chinese version of the scale was cross-culturally adapted and validated by Hu et al. (4) in a sample of 499 clinical nurses in Sichuan Province, demonstrating good psychometric properties.The scale comprises 15 items distributed across two subscales: positive attitude (9 items) and negative attitude (6 items). The positive attitude subscale reflects a tendency to recognize and accept the benefits, value, and future prospects of AI in nursing, whereas the negative attitude subscale captures concerns and unease regarding potential risks, ethical issues, and professional impacts associated with AI applications.All items are rated on a 5-point Likert scale ranging from 1 (strongly disagree) to 5 (strongly agree). The total score ranges from 15 to 75, with a theoretical midpoint of 45; higher scores indicate more positive attitudes toward the application of AI technologies. In the original validation study, the overall Cronbach's *α* was 0.910. In the present study, the Cronbach's *α* for the total scale was 0.845.

### Artificial intelligence literacy scale (AILS)

The Artificial Intelligence Literacy Scale (AILS), developed by Wang et al., was used to assess AI literacy in the general population (6). The scale consists of 12 items distributed across four dimensions: Awareness, Use, Evaluation, and Ethics, with each dimension containing 3 items.Responses are recorded on a 7-point Likert scale ranging from 1 (strongly disagree) to 7 (strongly agree). The total score ranges from 12 to 84, with higher scores indicating a higher level of AI literacy. In a previous study involving Turkish surgical nurses, the scale demonstrated good internal consistency with a Cronbach's *α* coefficient of 0.821 (8). In the present study, the Cronbach's α coefficient for the AILS was 0.858.

### The six-item change fatigue measurement scale

The Six-item Change Fatigue Measurement Scale, originally developed by Beernerth et al. and cross-culturally adapted into Chinese by Zhang Xinyue et al., was used to measure change fatigue levels among clinical nurses who had experienced organizational changes in hospital environments (8).The scale consists of 6 items, each rated on a 7-point Likert scale ranging from 1 (strongly disagree) to 7 (strongly agree). The total score ranges from 6 to 42, with higher scores indicating more severe change fatigue. In the original validation study, the scale demonstrated good internal consistency with a Cronbach's α coefficient of 0.85. In the present study, the Cronbach's α coefficient for this scale was 0.910.

### Data collection

Data were collected via electronic questionnaires distributed through WeChat. A QR code linking to the questionnaire was disseminated to nursing staff in clinical departments. The preface of the questionnaire clearly outlined the study purpose, instructions for completion, and privacy protection measures. Participation was voluntary, and informed consent was obtained from all participants before they proceeded to complete the questionnaire.To ensure data completeness, the questionnaire incorporated a mandatory response feature, which prevented submission until all items were answered and provided real-time reminders for any unanswered questions. An anonymous survey mechanism was implemented, and technical measures were applied to restrict each user to a single submission, thereby eliminating duplicate responses. Invalid questionnaires—defined as those with a completion time of less than 120 s or showing patterned responses—were excluded. A total of 468 questionnaires were returned. After exclusion of invalid responses, 449 valid questionnaires were retained, yielding an effective response rate of 95.94%.

### Ethical considerations

This study was approved by the Ethics Committee of Rudong Hospital (Approval No. 2022001). The study was conducted in accordance with the Declaration of Helsinki and its subsequent amendments or similar ethical standards. All participants signed an informed consent form and were given an explanation of the study's importance and goal by the researcher before the official investigation. Participants were told there would be no consequences if they chose to leave the research at any point. The researcher pledged to maintain the complete confidentiality and anonymity of the participants' personal information.

### Statistical analysis

Data analysis was performed using SPSS 26.0. Descriptive statistics were computed for all variables. Categorical data were presented as frequencies and percentages. Continuous variables with normal distribution were expressed as mean ± standard deviation, while non-normally distributed data were presented as median and interquartile range.

To enable direct comparison across the different measurement scales used in this study, the percentage of the maximum possible score was calculated for outcomes. This percentage was derived for each scale or subscale using the formula: (Mean Score/Theoretical Maximum Score) × 100%, where the Theoretical Maximum Score was defined by the structure and scoring system of each original instrument.

Independent samples t-tests and one-way ANOVA were used to compare AI attitude scores across different demographic characteristics. Variables showing statistical significance in univariate analysis (*P* < 0.05) were included as independent variables in multiple linear regression analysis to identify factors influencing AI attitudes. The reliability of the scales used in this study was assessed using Cronbach's α coefficient, with values above 0.70 considered acceptable. The significance level was set at *P* < 0.05 for all statistical tests.

## Results

### Current status of AI attitudes, AI literacy, and change fatigue

A total of 450 nurses from county-level hospitals participated in this study. The overall score for AI application attitudes was 45.17 ± 2.38, falling within the moderate range of the theoretical scale (15–75 points) and representing 60.23% of the maximum possible score.

Analysis of the attitude dimensions revealed an important pattern. The positive attitude subscale score was 22.99 ± 5.59 (51.09% of its maximum score of 45). In comparison, the negative attitude subscale score was 22.18 ± 3.78, achieving a significantly higher percentage of its maximum possible score at 73.93% (based on a maximum of 30 points). This indicates that negative perceptions represent a more prominent aspect of the overall attitude structure than positive perceptions among this cohort.

Regarding AI literacy, the mean total score on the AILS was 50.02 ± 4.67 (59.55% of the maximum score of 84). Among the subdimensions, Awareness received the highest mean score (12.98 ± 1.36, 61.81% of 21 points), followed by Ethics (12.54 ± 1.15, 59.71% of 21 points), Evaluation (12.44 ± 1.16, 59.24% of 21 points), and Use (12.06 ± 1.13, 57.43% of 21 points).

Additionally, the mean score for Change Fatigue was 30.88 ± 7.82 (73.52% of the maximum score of 42), indicating a moderate to high level of organizational change fatigue among participants. Detailed score distributions and mean scores per item for all variables are presented in Table 1.

**Table 1:** Scores of county-level clinical nurses’ attitudes toward AI applications, AILS, and change fatigue (*n* = 449, $\bar{X}\pm S$).

Subject	Minimum value	Maximum value	Total mean score	Percentage of Max Score (%)
Positive attitude dimension	10	38	22.99+−5.59	51.09
Negative attitude dimension	10	30	22.18+−3.78	73.93
Total Score of AI Application Attitude	38	51	45.17+−2.38	60.23
Consciousness dimension	9	16	12.98+−1.36	61.81
Using dimensions	9	15	12.06+−1.13	57.43
Evaluation dimension	9	16	12.44+−1.16	59.24
Ethical dimension	9	16	12.54+−1.15	59.71
AILS	36	63	50.02+−4.67	59.55
Change fatigue	13	46	30.88+−7.82	73.52

### Comparison of AI literacy scores by demographic characteristics

See Table 2.

**Table 2:** Comparison of AI technology application attitude scores among county-level clinical nurses (*n* = 449, $\bar{X}\pm S$).

Subject		*N*	AI application attitude score	*t/F*	*P*
Gender	Male	2	45.73+−0.78	0.333	0.739
	Female	447	45.17+−2.39		
Age (years)	≤25	70	44.33+−2.09	17.104	**<0**.**001**
	26–30	126	44.28+−1.79		
	31–40	191	45.83+−2.57		
	>40	62	45.88+−2.31		
Educational background	Secondary specialized school	139	44.26+−2.07	227.176	**<0**.**001**
	Junior college	234	44.5+−1.67		
	Bachelor's degree or higher	76	48.91+−0.5		
Years of service	≤3	137	44.32+−2.12	38.352	**<0**.**001**
	4–6	82	44.15+−1.43		
	7–10	70	44.32+−1.76		
	11–15	65	46.9+−2.38		
	≥15	95	46.71+−2.38		
Professional Title	Junior professional title and below	253	44.33+−1.82	61.686	**<0**.**001**
	Intermediate professional title and below	184	46.04+−2.51		
	Senior professional titles and below	12	49.58+−0		
Monthly income(RMB)	≤4,000	79	44.03+−1.66	52.682	**<0**.**001**
	4,001–6,000	150	44.31+−1.88		
	6,001–8,000	72	44.68+−1.69		
	≥8,000	148	46.88+−2.52		
Number of night shifts per month (unit)	0	98	46.55+−2.51	16.007	**<0**.**001**
	1–3	44	45.2+−1.73		
	4–9	287	44.72+−2.3		
	≥10	20	44.85+−1.45		
AI-related knowledge training	Have	54	48.15+−0.96	19.727	**<0**.**001**
	None	395	44.76+−2.22		

Bold values indicate statistical significance at *P* < 0.05.

### Multivariate analysis of factors influencing AI attitudes among county-level clinical nurses

Multiple linear regression analysis was performed with the total score of AI application attitudes as the dependent variable and variables showing statistical significance in univariate analysis as independent variables. The results revealed that age, participation in AI-related training, education level, number of monthly night shifts, change fatigue, and AILS score were significant factors influencing AI attitudes among county-level clinical nurses (*P* < 0.05). Collectively, these factors accounted for 60.7% of the total variance in AI attitude scores.

Among these influencing factors, change fatigue (β' = −0.346) and AILS total score (β' = 0.271) emerged as the two most influential predictors, followed by training (β' = −0.111), education level (β’ = 0.123), number of monthly night shifts (β' = −0.108), and age (β' = −0.108). The regression model was statistically significant (*F* = 77.527, *P* < 0.001). Detailed results are presented in Table 3.

**Table 3:** Multiple linear regression analysis of influencing factors on county-level clinical nurses’ attitudes toward AI applications (*N* = 449).

Subject	β	Standard error	β’	*t*	*P*
(Constant)	42.918	1.436		29.89	0
Age	−0.281	0.134	−0.108	−2.094	**0**.**037**
Educational background	0.432	0.15	0.123	2.87	**0**.**004**
Professional Title	0.127	0.22	0.029	0.578	0.563
Monthly income	0.057	0.092	0.027	0.623	0.534
Number of night shifts per month	−0.292	0.104	−0.108	−2.814	**0**.**005**
AI-related knowledge training	−0.808	0.293	−0.111	−2.761	**0**.**006**
Length of service	0.153	0.084	0.098	1.825	0.069
Change fatigue	−0.105	0.014	−0.346	−7.372	**0**
AILS	0.138	0.021	0.271	6.602	**0**

*R*^2^ = 0.614, adjust *R*^2^ = 0.606; *F* = 77.527, *P* < 0.001.

Bold values indicate statistical significance at *P* < 0.05.

## Discussion

### AI attitude scores among county-level clinical nurses

The findings of this study indicate that the total AI application attitude score among county-level clinical nurses was 45.17 ± 2.38, slightly above the theoretical median of the scale (45 points), indicating an overall moderate level. However, this score was significantly lower than those reported by Hu et al. (4) and Li Chuang et al. (3) in studies involving nurses from tertiary hospitals. This discrepancy may be attributed to the fact that the participants in this study were from Chinese county-level hospitals, suggesting that the level of healthcare institutions and differences in resource environments within the Chinese healthcare system may significantly influence nurses' willingness to adopt AI technologies.

The slightly higher score in the negative attitude dimension compared to the positive attitude dimension reflects that while nurses acknowledge the potential value of AI, they also harbor more prominent concerns, unease, and skepticism. This indicates that county-level nurses are currently in a “cognitive-affective contradiction” stage regarding AI technology adoption. This phenomenon may stem from their insufficient understanding of AI principles and boundaries, implicit anxiety about role reconstruction, and uncertainty regarding their ability to adapt to digital workflow transformations (9).

In summary, the neutral, contradictory, yet malleable attitudes of county-level clinical nurses toward AI applications present both a potential opportunity for advancing intelligent nursing and a practical implementation risk. Healthcare institution managers should seize this critical window by implementing comprehensive strategies, such as establishing systematic training systems to enhance AI literacy, promoting transparent communication to resolve cognitive biases, and rationally allocating human resources to alleviate change fatigue. These measures can gradually guide this group from “cautious observation” to “rational acceptance”, laying the necessary psychological and organizational foundation for the orderly integration of AI technology in primary care settings. The key determinants of AI attitudes identified in this study, along with their interpretations and practical implications, are synthetically summarized in Table 4.

**Table 4:** Summary of Key findings, interpretations, and practical recommendations.

Key finding	Plausible interpretation/cause	Recommendations for practice & policy
Moderate, ambivalent overall AI attitude	Cognitive-affective contradiction; recognition of value coupled with prominent concerns.	Implement transparent communication about AI's role and boundaries; address ethical concerns proactively.
Younger age → Positive attitude	Greater exposure and adaptability as “digital natives".	Leverage younger nurses as peer mentors; tailor training for different age groups.
Low AI training coverage (12%)	Systemic shortage of structured training resources in county settings.	Formally integrate practical, clinically-relevant AI training into nursing continuing education systems.
Higher education → Positive attitude	Enhanced information literacy, learning capacity, and critical evaluation skills.	Consider educational background in talent strategies; establish mentorship from higher-educated nurses.
More night shifts → Negative attitude	Cognitive fatigue and emotional exhaustion reducing capacity for new learning.	Optimize scheduling to reduce night shift burden; provide supportive learning environments for affected staff.
Higher AI literacy → Positive attitude	Better understanding reduces anxiety and builds technical self-efficacy.	Develop systematic AI literacy programs; link literacy development to professional advancement.
Higher change fatigue → Negative attitude	Psychological exhaustion from ongoing organizational changes reduces openness.	Adopt proactive change management strategies; space out major initiatives to mitigate fatigue.

### Factors influencing AI attitudes among county-level clinical nurses

#### Younger age predicts more positive attitudes toward AI

This study identified age as a significant negative predictor of nurses' attitudes toward AI, indicating that younger nurses generally demonstrate higher acceptance of emerging technologies compared to their older counterparts. This generational difference may be attributed to several factors: younger nurses, having grown up as “digital natives” with greater exposure to information technology, tend to possess an inherent affinity for innovative technologies like AI. In contrast, older nurses, with more established work habits and potentially lower technological adaptability, may experience greater adaptation anxiety when confronting technological transformations (10).

Notably, the nursing workforce in the studied county-level hospitals demonstrated a characteristic middle-aged distribution, with nurses aged 31 years and older comprising 56.43% of the sample. This demographic profile may further amplify the negative impact of age on AI attitudes. Given that Chinese county-level healthcare institutions commonly face challenges such as an aging nursing workforce and relatively delayed technological updates, we recommend implementing stratified training strategies with differentiated programs tailored to the characteristics of different age groups (11).

For older nurse cohorts, training should focus on reducing technological barriers through targeted one-on-one guidance and practical instruction. Simultaneously, healthcare institutions should leverage the technical leadership of younger nurses by establishing peer support mechanisms, thereby comprehensively enhancing the overall technology acceptance level across the nursing team (12).

#### AI training as a significant predictor of positive attitudes

This study identified prior artificial intelligence training as a significant positive predictor of nurses' AI application attitudes, a finding consistent with multiple domestic and international studies (13–15). For instance, Shokr A et al. (15) demonstrated that incorporating a knowledge-based AI chatbot into nursing training programs resulted in statistically significant knowledge improvement among nurses. Similarly, Yıldırım Gürkan D et al. (13) through interviews with 20 nursing students, found considerable anticipation for AI-related education.

Training likely influences attitudes through two primary mechanisms: systematically enhancing nurses' understanding of AI technologies and building application confidence, thereby reducing technology anxiety stemming from knowledge gaps (16); and facilitating rational cognition through hands-on practice and case discussions during training, helping overcome resistance arising from unfamiliarity (17).

Notably, data revealed that only 54 nurses (12.0%) in the studied county hospital had received AI-related training, indicating notably low training coverage and highlighting a systemic shortage of training resources in such settings. We therefore recommend that county healthcare institutions formally integrate AI training into nursing continuing education systems, prioritizing the development of practical training curricula aligned with frontline clinical needs. Utilizing diverse formats such as workshops and case studies could enhance training effectiveness, thereby establishing a necessary foundation for successful AI implementation in county-level medical institutions.

#### Higher education level predicts more positive AI attitudes

This study identified education level as a significant positive predictor of nurses' attitudes toward AI applications, indicating that within the county hospital context, nurses with higher educational backgrounds demonstrate more favorable dispositions toward AI technologies. This relationship may be explained by several factors: advanced education cultivates stronger information literacy and learning capabilities, enabling nurses to more readily comprehend and master the fundamental principles and practical applications of AI (18). Furthermore, systematic academic training enhances critical thinking and evaluation skills regarding emerging technologies, contributing to the development of more rational and informed perspectives (19).

In the environment of county-level medical institutions, which often face relative shortages of specialized technical talent and slower technological updates, highly educated nurses represent a crucial force for driving technological innovation. It is recommended that healthcare organizations strategically consider educational background in both talent recruitment and development programs to optimize workforce structure. Additionally, establishing a mentorship model where highly educated nurses guide their less-educated colleagues can maximize technological leadership. This could be implemented through forming AI technology learning groups and organizing thematic workshops to collectively enhance the technical proficiency of the entire nursing team. Furthermore, developing differentiated training programs tailored to nurses with varying educational backgrounds will ensure that training content aligns with their respective cognitive levels and learning needs.

#### Lower frequency of night shifts predicts more positive AI attitudes

The results identified the number of monthly night shifts as a significant negative predictor of nurses' AI application attitudes, indicating that greater night shift burden is associated with less favorable dispositions toward AI technology among nurses in county-level hospitals. Potential mechanisms underlying this relationship include the chronic fatigue state induced by frequent night shifts, which depletes the cognitive resources and mental energy necessary for learning new technologies (20). Concurrently, irregular work schedules may contribute to emotional exhaustion, thereby reducing nurses' capacity to adapt to workplace changes (21).

This effect is particularly pronounced in county-level medical institutions, where human resources are often constrained and nursing workloads are generally heavy. To mitigate this barrier, healthcare institutions should optimize scheduling systems and rationally allocate human resources to reduce nurses' night shift burden. For nurses who must undertake a higher frequency of night shifts, more supportive learning environments should be provided, including flexible training schedules and access to personalized learning resources (22). Furthermore, integrating AI training with supportive measures such as fatigue management and psychological adjustment strategies could comprehensively enhance nurses' willingness and ability to adopt new technologies across multiple dimensions (23).

#### Higher AI literacy predicts more positive attitudes toward AI

Artificial intelligence literacy emerged as a significant positive predictor of nurses' AI application attitudes in the multiple linear regression analysis. This finding indicates that within the county hospital setting, nurses with higher levels of AI literacy demonstrate more favorable attitudes toward AI technologies. This result aligns with the conclusions of Kong Hanhan et al. (6), whose research emphasized that enhancing AI literacy among healthcare providers represents a crucial pathway for improving technology acceptance.

The relationship between AI literacy and positive attitudes can be explained through several mechanisms. First, greater AI literacy enables nurses to more accurately comprehend AI technology's operational principles and application boundaries, thereby reducing technology anxiety stemming from knowledge gaps (24). Second, a solid foundation in AI knowledge enhances nurses' confidence in utilizing AI tools in clinical practice, strengthening their technical self-efficacy (25).

Given the constrained technical resources and limited training opportunities typically available in county-level medical institutions, enhancing nurses' AI literacy becomes particularly crucial for facilitating successful technology implementation. We recommend that healthcare institutions establish systematic AI literacy enhancement programs, incorporating fundamental AI knowledge, technological principles, ethical guidelines, and application skills into the core content of nursing continuing education. Furthermore, developing incentive mechanisms that link AI literacy with professional advancement could effectively encourage nurses to proactively develop these essential competencies.

#### Lower change fatigue predicts more positive AI attitudes

Multiple linear regression analysis identified change fatigue as the most substantial negative predictor of nurses' AI application attitudes (β' = −0.346, *P* < 0.001), indicating that nurses experiencing lower levels of change fatigue demonstrated significantly more positive attitudes toward AI technologies. This finding aligns with existing evidence demonstrating that psychological exhaustion from persistent organizational changes substantially reduces healthcare professionals' willingness to adopt new technologies (26).

The context of county-level hospitals is particularly relevant, as these institutions have recently undergone sequential major organizational transformations, including facility relocations and electronic health record implementations, resulting in elevated levels of change fatigue among medical staff (21). Research indicates that sustained psychological depletion fundamentally undermines both the willingness and capacity to embrace new challenges (27). Within this context, repeated organizational changes may induce emotional desensitization and change resistance among county-level nurses, consequently fostering a defensive stance toward emerging technologies such as AI.

To mitigate this significant barrier, healthcare institutions should implement systematic change management strategies that carefully regulate the pace of technological introductions and process modifications. This approach would prevent the cumulative burden of simultaneous changes and reduce fatigue accumulation. We further recommend providing adequate psychological support and resources during transition periods to facilitate adaptation and maintain openness to technological innovations.

## Limitations

This study has several limitations that should be considered when interpreting the findings. First, the data were collected from a single county-level hospital in Eastern China's Jiangsu Province, which may limit the generalizability of the findings to hospitals in other Chinese regions with different socioeconomic and healthcare contexts or to other countries. Second, the sample exhibited limited diversity in gender distribution, with female nurses comprising the vast majority (99.6%) of participants, potentially limiting the understanding of gender-specific perspectives on AI adoption. Additionally, the cross-sectional design precludes the establishment of causal relationships between the identified factors and AI attitudes. While our regression model explained a substantial portion of the variance in AI attitudes, other potentially relevant variables—such as organizational culture, leadership support, and specific AI exposure experiences—were not measured and thus remain unexplored. Future research would benefit from multi-center studies across diverse geographic locations, purposive sampling to ensure better gender representation, longitudinal designs to track attitude evolution, and the inclusion of additional organizational and experiential variables to provide a more comprehensive understanding of the factors influencing AI acceptance among county-level hospital nurses.

## Conclusion

This study revealed that clinical nurses in county-level hospitals generally hold moderate attitudes toward AI applications, influenced by a complex interplay of factors. Nurses with AI-related training, higher education levels, and greater AI literacy demonstrated more positive attitudes toward AI technologies. Conversely, older age, more frequent night shifts, and higher levels of change fatigue were associated with less favorable attitudes toward AI implementation.

## Data Availability

The raw data supporting the conclusions of this article will be made available by the authors, without undue reservation.

## References

[1] Yıldırım T Karaman M. Development and psychometric evaluation of the artificial intelligence attitude scale for nurses. BMC Nurs. (2025) 24(1):441. 10.1186/s12912-025-03098-6 40264200 PMC12013020

[2] Chen Z Wu T Wu X Wang J Ke S Li H The mediating effects of technology trust and perceived value in the relationship between eHealth literacy and attitude toward the usage of artificial intelligence in nursing: a cross-sectional study. BMC Nurs. (2025) 24(1):989. 10.1186/s12912-025-03577-w 40731339 PMC12309084

[3] Li C Guo H Zhang L. Translation and validation of the Chinese version of the attitude scale for the application of homo sapiens artificial intelligence technology in the nursing field. Military Nurs. (2025) 42(10):6–9. 10.3969/j.issn.2097-1826.2025.10.002

[4] Hu R Ma Y-f Wang C-y Li X-y Huang H-p. Validity and reliability of the Chinese translation of the attitude scale towards the use of artificial intelligence technologies in nursing (ASUAITIN)- a cross-sectional study. BMC Nurs. (2025) 24(1):782. 10.1186/s12912-025-03322-3 40597054 PMC12211220

[5] Yixin T Jianxing Y. County medical community reform: china’s solution for building an integrated healthcare service system with broussonetia papyrifera. Gov Stud. (2025) 41(01):81–90+159. 10.15944/j.cnki.33-1010/d.2025.01.002

[6] Hanhan K Youfeng S Huanhuan Ps Ganggang P Jing C Min W. A study on the current status and influencing factors of artificial intelligence literacy among clinical nurses. J Nurs Sci. (2025) 40(18):106–9. 10.3870/j.issn.1001-4152.2025.18.106

[7] Yılmaz D Uzelli D Dikmen Y. Psychometrics of the attitude scale towards the use of artificial intelligence technologies in nursing. BMC Nurs. (2025) 24(1):151. 10.1186/s12912-025-02732-7 39930420 PMC11809082

[8] Xinyue Z Mengting P Ying W Shiyu Y Yue G Zijing H. Translation and validation of the change fatigue scale. Chin Nurs Res. (2024) 38(24):4387–91. 10.12102/j.issn.1009-6493.2024.24.009

[9] Özçevik Subaşi D Akça Sümengen A Semerci R Şimşek E Çakır GN Temizsoy E. Paediatric nurses’ perspectives on artificial intelligence applications: a cross-sectional study of concerns, literacy levels and attitudes. J Adv Nurs. (2025) 81(3):1353–63. 10.1111/jan.16335 39003632

[10] Labrague LJ Aguilar-Rosales R Yboa BC Sabio JB de los Santos JA. Student nurses’ attitudes, perceived utilization, and intention to adopt artificial intelligence (AI) technology in nursing practice: a cross-sectional study. Nurse Educ Pract. (2023) 73:103815. 10.1016/j.nepr.2023.103815 37922736

[11] Chiu P-W Chu S-C Yang C-H Lee H-F Hung H-M Hsu H-C. Peer-assisted learning in critical care: a simulation-based approach for postgraduate medical training. Med Educ Online. (2025) 30(1):2497333. 10.1080/10872981.2025.2497333 40325999 PMC12057776

[12] Mollet MC Zerhouni O Bagnis CI Romo L. Preliminary clustering: exploring the interplay of burnout, stress, turnover, psychological flexibility and distress in a French nurse sample. J Adv Nurs. (2025). 10.1111/jan.70299 41145353 PMC13176726

[13] Yıldırım Gürkan D Çakmak R. Nursing students metaphorical perceptions of the concept of “Artificial intelligence” and their views on the future of artificial intelligence. BMC Nurs. (2025) 24(1):1275. 10.1186/s12912-025-03947-4 41088190 PMC12522490

[14] Bodur G Cakir H Turan S Seren AKH Goktas P. Artificial intelligence in nursing practice: a qualitative study of nurses’ perspectives on opportunities, challenges, and ethical implications. BMC Nurs. (2025) 24(1):1263. 10.1186/s12912-025-03775-6 41088159 PMC12522738

[15] Shokr EA. Integrating a knowledge-based artificial intelligence chatbot into nursing training programs: a comparative quasi-experimental study in Egypt and Saudi Arabia. BMC Nurs. (2025) 24(1):1245. 10.1186/s12912-025-03883-3 41063074 PMC12505844

[16] El Arab RA Al Moosa O Sagbakken M Ghannam A Abuadas FH Somerville J Integrative review of artificial intelligence applications in nursing: education, clinical practice, workload management, and professional perceptions. Front Public Health. (2025) 13:1619378. 10.3389/fpubh.2025.1619378 40823249 PMC12354398

[17] Chan MMK Wan AWH Cheung DSK Choi EPH Chan EA Yorke J Integration of artificial intelligence in nursing simulation education: a scoping review. Nurse Educ. (2025) 50(4):195–200. 10.1097/NNE.0000000000001851 40168182

[18] Arabfard M Jeffery AD Moradian S He H-G Pandian V Vahedian-Azimi A. The ideal human care in green ICU: an integrated AI framework for future ICU care. Intensive Crit Care Nurs. (2025) 91:104213. 10.1016/j.iccn.2025.104213 40897580

[19] Clement David-Olawade A Wada OZ Adeniji YJ Aderupoko IV Olawade DB. Artificial intelligence readiness among healthcare students in Nigeria: a cross-sectional study assessing knowledge gaps, exposure, and adoption willingness. Int J Med Inform. (2025) 204:106085. 10.1016/j.ijmedinf.2025.106085 40845524

[20] Sumner J Shankar R Bundele A Yap A Ali JM Teng GG Insights from high and low clinical users of telemedicine: a mixed-methods study of clinician workflows, sentiments, and user experiences. Int J Med Inform. (2025) 203:106044. 10.1016/j.ijmedinf.2025.106044 40674858

[21] Yu M Wang H Wu Y Zhang Q Du X Huang X The influence of emotional intelligence on psychiatric Nurses’ care behavior, and the chain mediating role of compassion fatigue and perception of management. J Psychosoc Nurs Ment Health Serv. (2025) 63(2):35–43. 10.3928/02793695-20241101-02 39508679

[22] Alshammari MH Alboliteeh M. The mediating role of nurses' spiritual well-being between moral resilience and compassion fatigue: a multicenter structural equation model study. Int Nurs Rev. (2025) 72(1):e13082. 10.1111/inr.13082 39690492

[23] Silva-Ferreira M Cruz S Luís MS Silva MC Monteiro-Reis S Henrique R Mapping study on AI-based technologies in palliative care—a scoping study. BMC Palliat Care. (2025) 24(1):274. 10.1186/s12904-025-01909-w 41152966 PMC12570659

[24] Jiang Z Liu Q Jiang N Ning M Yu Q. Artificial intelligence literacy and its associated factors among nursing students. Nurse Educ. (2025) 50(6):E378–83. 10.1097/NNE.0000000000001989 40966073

[25] Al-Qerem W Jarab A Eberhardt J Abdo S Al-sa’di L Al-Shehadeh R Medication adherence among Jordanian adults with chronic conditions: a combined analysis using regression and machine learning. Ann Med. (2025) 57(1):2548979. 10.1080/07853890.2025.2548979 40833816 PMC12369517

[26] Yu M Ji J Kan D Gu D Yin X Cai S Analysis of the current situation and influencing factors of nurse change fatigue. Front Public Health. (2025) 13:1566534. 10.3389/fpubh.2025.1566534 40270743 PMC12016666

[27] Zhou C Huang X Yu T Wang C Jiang Y. Effects of compassion fatigue, structural empowerment, and psychological empowerment on the caring behaviours of intensive care unit nurses in China: a structural equation modelling analysis. Aust Crit Care. (2025) 38(3):101166. 10.1016/j.aucc.2024.101166 40054015

